# May bullous pemphigoid be worsened by COVID-19 vaccine?

**DOI:** 10.3389/fmed.2022.931872

**Published:** 2022-11-01

**Authors:** Emanuele Cozzani, Giulia Gasparini, Roberto Russo, Aurora Parodi

**Affiliations:** ^1^Section of Dermatology, Department of Health Sciences (DISSAL), University of Genoa, Genoa, Italy; ^2^Dermatology Unit, Ospedale Policlinico San Martino, Genoa, Italy

**Keywords:** bullous pemphigoid, COVID-19, vaccines, COVID vaccines, cutaneous reactions to vaccines

The claim implying that vaccines may cause autoimmune diseases has been extensively reported even before the advent of COVID-19. With the universal anti-SARS-CoV-2 immunization campaign, claims of the onset or relapses of autoimmune diseases after vaccination started to increase. This phenomenon did not spare cutaneous conditions, such as autoimmune bullous diseases (AIBDs). Special attention was dedicated to vaccine-induced bullous pemphigoid (BP). However, this association is currently debated.

Notably, COVID-19 *per se* does not seem to be correlated to BP induction, only two cases have been reported in the literature (1, 2).

Up to date 51 cases of bullous pemphigoid (BP) after COVID-19 vaccination have been reported in the literature, as single case reports or as small case series. However, in 2 of the 51 reported cases, the patients were simultaneously exposed to another known trigger of BP, namely gliptine therapy (3, 4). Moreover, a recently published cohort study on large scale (more than 1.000.000 persons), found no relationship between *de novo* development of bullous pemphigoid and mRNA COVID-19 vaccination (5). Nonetheless, as these Authors state, their study only reports on the risk of new-onset bullous pemphigoid and does not offer insight as to whether vaccination can flare or cause an exacerbation of the disease.

Thus, based on the existing literature, it seems quite improbable that COVID-19 or anti-COVID-19 vaccines can easily induce BP *de novo*. Nonetheless, anti-COVID-19 vaccines could be a trigger contributing together with others that may exacerbate a disease flare.

Indeed, reports have been published of patients with BP experiencing recurrences after vaccination, during a period of remission. Up to date, 6 cases of flares in patients who were previously diagnosed of BP were published (Table 1). In our clinical practice we only witnessed few cases of BP flares after COVID-19 vaccination.

**Table 1:** Published cases of flares after COVID-19 vaccination in patients who were previously diagnosed of BP.

**No**.	**Gender**	**Age (years)**	**Vaccine**	**After which dose**	**Latency from last dose (days)**	**DIF**	**IIF**	**ELISA anti-BP180 (U/mL)**	**ELISA anti-BP230 (U/mL)**	**References**
1	F	75	Moderna	1st	3	+	+	ND	ND	Damiani, (9)
2	M	84	Moderna	1st	14	+	+	ND	ND	
3	F	82	Pfizer	1st	3	+	+	ND	ND	
4	F	74	CoronaVac	1st	7	+	ND	ND	ND	Afacan, (10)
5	F	65	CoronaVac	2nd	7	+	ND	ND	ND	
6	M	71	CoronaVac	2nd	45	+	ND	ND	ND	

ND, not determined.

Considering the cases of BP relapses reported by the literature, the latency period from COVID-19 vaccination and the BP flare was extremely variable ranging from 3 to 45 days after the last dose. Thus, the BP flares do not seem to follow any specific temporal pattern.

The possible pathogenic mechanisms underlying of BP flares could be the same that have been hypothesized for BP induction *de novo*. Proposed mechanisms involve non-specific bystander immune activation, molecular mimicry, and in the context of the COVID-19 pandemic, a novel consequence of mRNA vaccine technology. A first hypothetic mechanism implies a molecular mimicry, between specific basement membrane proteins (e.g., BP-180, BP-230) and the spike protein of SARS-CoV-2 (6); although, the exposure to native spike protein during SARS-CoV-2 infection doesn't seem to be a triggering factor for BP, therefore the exposure to the immune system of spike protein antigens through vaccination appears to be an unlikely triggering mechanism. A second hypothesis that susceptible individuals with a pre-existing autoimmune/autoinflammatory dysregulation may have an increased risk of immunological side effects after administration of such vaccines, some of which contain nucleic acids. According to this hypothesis, in pre-translationally predisposed individuals, mRNA vaccines might trigger different pro-inflammatory pathways through binding to Toll-like receptors (TLR) −3, −7, and −8 (7). Moreover, through cytokine modulation, novel antigens and adjuvants could promote T-cell-dependent immune responses that would lead to the production of autoreactive B cells; supporting this mechanism, T-cell clones reactive to SARS-CoV-2 were found in the skin infiltrate of two patients with BP induced by COVID-19 vaccination (6). The latter two hypotheses, could also explain another pathogenic pathway, namely that in patients undergoing polypharmaceutical therapy, with potentially PB-inducing drugs (antihypertensives, salicylates, and diuretics), anti-SARS-CoV-2 vaccines may create an immune environment suitable for developing drug-induced PB (8). This could be a way of indirectly inducing BP flares. However, none of these pathogenetic mechanisms are completely convincing and have not been thoroughly supported by scientific evidence.

Moreover, it is widely known that BP is a chronic illness that may present frequent relapses without a specific triggering agent or that occur simply during steroid tapering. Indeed, in our clinical practice we have not noticed an increase in the number of relapses in BP since the beginning of the vaccination campaign.

In the last 2 years of the pandemic, the presence of skin reactions after COVID-19 and post COVID-19 vaccination might have led clinicians, unconsciously or consciously, to excessively investigate and find a possible relationship between the new-onset skin manifestations and a previous COVID-19 vaccination. This might explain the publications on this topic.

Further studies are needed to better investigate whether COVID-19 vaccines may really induce BP flares, but with the current knowledge this association doesn't seem to be true. In conclusion, in the unprecedented scenario of a universal vaccination, it is likely that some cases of bullous pemphigoid that would have relapsed spontaneously occurred in temporal coincidence with the vaccination.

## Author contributions

EC, GG, and RR wrote the first draft of the manuscript. All authors wrote sections of the manuscript and contributed to manuscript revision, read, and approved the submitted version.

## Conflict of interest

The authors declare that the research was conducted in the absence of any commercial or financial relationships that could be construed as a potential conflict of interest.

## Publisher's note

All claims expressed in this article are solely those of the authors and do not necessarily represent those of their affiliated organizations, or those of the publisher, the editors and the reviewers. Any product that may be evaluated in this article, or claim that may be made by its manufacturer, is not guaranteed or endorsed by the publisher.
